# Fluconazole-loaded solid lipid nanoparticles topical gel for treatment of pityriasis versicolor: formulation and clinical study

**DOI:** 10.1080/10717544.2017.1413444

**Published:** 2017-12-14

**Authors:** Shaimaa El-Housiny, Maii Atef Shams Eldeen, Yasmina Ahmed El-Attar, Hoda A. Salem, Dalia Attia, Ehab R. Bendas, Mohamed A. El-Nabarawi

**Affiliations:** ^a^ Department of Pharmaceutics and Industrial Pharmacy, Faculty of Pharmacy, Modern University for Technology and Information Cairo Egypt; ^b^ Department of Microbiology, Faculty of Medicine, Tanta University Tanta Egypt; ^c^ Department of Dermatology and venereology, Faculty of Medicine, Tanat University Tanta Egypt; ^d^ Department of Clinical Pharmacy, Faculty of Pharmacy, Al-Azhar University Cairo Egypt; ^e^ Department of Pharmaceutics and Industrial Pharmacy, Faculty of Pharmacy, The British University in Egypt (BUE) Cairo Egypt; ^f^ Department of Pharmaceutics and Industrial Pharmacy, Faculty of Pharmaceutical Sciences and Pharmaceutical Industries, Future University in Egypt Cairo Egypt; ^g^ Department of Pharmaceutics and Industrial Pharmacy, Faculty of Pharmacy, Cairo University Cairo Egypt

**Keywords:** Fluconazole, solid lipid nanoparticles, topical delivery, entrapment efficiency, Carpabol 934, Pityriasis Versicolor, clinical study

## Abstract

Solid lipid nanoparticles (SLNs) are very potential formulations for topical delivery of antifungal drugs. Hence, the purpose of this research was to formulate the well-known antifungal agent Fluconazole (FLZ)-loaded SLNs topical gel to improve its efficiency for treatment of Pityriasis Versicolor (PV). FLZ-SLNs were prepared by modified high shear homogenization and ultrasonication method using different concentration of solid lipid (Compritol 888 ATO, Precirol ATO5) and surfactant (Cremophor RH40, Poloxamer 407). The physicochemical properties and the *in vitro* release study for all FLZ-SLNs were investigated. Furthermore, the optimized FLZ-SLN formula was incorporated into gel using Carpobol 934. A randomized controlled clinical trial (RCT) of potential batches was carried out on 30 well diagnosed PV patients comparing to market product Candistan^®^ 1% cream. Follow up was done for 4 weeks by clinical and KOH examinations. The results showed that FlZ-SLNs were almost spherical shape having colloidal sizes with no aggregation. The drug entrapment efficiency ranged from 55.49% to 83.04%. The zeta potential values lie between −21 and −33 mV presenting good stability. FLZ showed prolonged *in vitro* release from SLNs dispersion and its Carbapol gel following Higuchi order equation. Clinical studies registered significant improvement (*p* < .05) in therapeutic response (1.4-fold; healing%, 4-fold; complete eradication) in terms of clinical cure and mycological cure rate from PV against marketed cream. Findings of the study suggest that the developed FLZ loaded SLNs topical gels have superior significant fast therapeutic index in treatment of PV over commercially available Candistan^®^ cream.

## Introduction

Fluconazole (FLZ) is a third generation triazole antifungal drug with broad spectrum activity against systemic and superficial fungal infections (Ruhnke et al., 2008; Abdel-Mottaleb et al., 2009). It acts by preventing production of vital elements in the fungal membrane as ergosterol by inhibiting the fungal cytochrome P-450 enzyme (Nicky et al., 2008). This inhibition action on cytochrome P-450 enzymes was found to be greater in fungal species in contrast to mammalian enzyme, which improve the safety profile of triazoles (Koltin & Hitchcock, 1997). FLZ is slightly soluble in water (5 mg/mL at 37 °C), with molecular weight of 306.3 Da and has a p*K*_a_ value of 3.7 (weak base) (Alekha & William, 2006; Bidkar et al., 2007). It is presented as oral and parenteral dosage forms which can be associated with serious side effects as stomach upset, diarrhea, feeling sick, vomiting, rash, and reduction in red blood cells. In addition, there is incidence of hepatotoxicity in patients receiving triazoles (Song & Deresinski, 2005). Therefore, trying to develop topical pharmaceutical dosage forms of FLZ is highly indicated to avoid such adverse events (Gupta et al., 2010).

The drug delivery onto the skin is recognized as an effective means of local therapy for many dermatologic diseases (Kaur & Guleri, 2013). But the main obstacle facing the efficiency of topical antifungal drugs is the ability to penetrate the skin powerfully. Accordingly, the active material should pass in effective concentration through the stratum corneum, the first layer of the skin, to reach target tissue. Researchers may depend on pharmaceutical formulations as they exert a key role for permeation of drugs into skin. Development of novel approaches for local therapy of skin fungal infections utilizes advanced carrier systems for numerous compounds (Lee & Maibach, 2006). Nanoparticles are usually produced from natural/synthetic polymers with size range between 10 and 1000 nm. Therefore, they are suitable to optimize topical drug delivery and reduce toxicity (Mukherjee et al., 2009).

The nanoparticulate carrier systems as solid lipid nanoparticles (SLNs) has gained interest for the local therapy of skin fungal infection as they aid the permeation of loaded active materials through the stratum corneum layer (Gupta & Vyas, 2012). SLNs are colloidal carriers developed as an updating system to the ordinary used carriers as micro-emulsions and liposomes, as the liquid lipid (oil) has been substituted by a solid lipid. Hence, lots of unique properties have been linked to SLNs as small size, large surface area, high drug loading, and the interaction of phases at the interfaces, and improve performance of pharmaceuticals, neutraceuticals, and other materials (Cavalli et al., 1993).

Pityriasis versicolor (PV), also known as tinea versicolor, is a superfacial fungal infection caused by proliferation of Malassezia species in the stratum corneum layer of skin. Malassezia acquires a pathogenic potential when assuming the mycelial form under the influence of trigger factors including, humidity, immunosuppression, and hyperhidrosis (Karakas et al., 2005). Accordingly, PV is mostly common in humid weathers as compared to calm weathers (Crespo-Erchiga & Florencio, 2006). Indeed, the yeast most often becomes pathogenic in tropical climates, infecting up to 30–40% of the population in those areas (Faergemann, 1995). Cure rate from PV is hard, as relapse after treatment can be occur within 2 years (Faergemann, 1999). PV is presented with well-defined round or egg-shaped macules on the upper arms, trunk, and neck as the concentration of sebaceous glands is great; they can be white, erythematous, or brown (Mellen et al., 2004). PV appears frequently in adolescents (Dutta et al., 2002; Morais et al., 2010; Framil et al., 2011). The main concern for patients is their looking for treatment for the unpleasant appearance of the skin (Gupta et al., 2002). The topical antifungal agents are found to be quite satisfactory in the treatment of PV fungal infections, however, systemic antifungals are recommended for severe or recalcitrant cases (Hald et al., 2015). On the other hand, several non-specific topical treatments are used in treating PV (Gupta et al., 2004, 2005). Sometime, misdiagnosis may lead to wrong and unsuccessful treatment for example with antibiotics, corticosteroids (Mellen et al., 2004).

Improving the permeation of the antifungal agents to the dermis layer of skin is desired as fungi hyphae (mycelium) can enter deeply through the epidermal layers (Müller-Goymann, 2004). Thus, topical application of SLNs based gel with increased penetration and retention through skin because of lipid nano-formulation will be much promising for the topical treatment of fungal infections and symptomatic relief.

Therefore, the aim of the current study was to develop and to evaluate FLZ loaded-SLNs based gel for topical treatment of Pityriasis Versicolor. In addition, the golden goal of the research was to investigate clinically the efficacy of the prepared FLZ-SLNs gel compared to the antifungal topical formulation currently available in the market for topical treatment of patients with PV.

## Materials and methods

Flucoazole, Cremophor RH 40^®^ (HLB 14–16), Poloxamer 407 and Carbapol 934, were kindly supplied by Egyptian International Pharmaceutical Industries Company (E.I.P.I.Co) (10th of Ramadan City, Egypt). Compritol 888 ATO (glyceryl behenate) and pricerol ATO5 (glyceryl palmitostearate), were kindly gifted from Gattefosse Co. (Lyon, France). Absolute Ethanol, potassium dihydrogen phosphate and disodium hydrogen phosphate were purchased from Sigma-Aldrich Company (St. Louis, MO). All the other chemicals were of analytical grade.

### Preparation of solid lipid nanoparticles loaded with FLZ

The modified high shear homogenization and ultrasonication method has been used for preparation of eight formulae of FLZ-SLNs (Priyanka & Sathali, 2012). Lipids (Compritol 888 ATO or Precirol ATO5) were heated by 50 °C above their melting points. Then after 1% w/w FLZ was added to the lipid matrix to obtain a drug-lipid mixture. While the aqueous phase was obtained by dissolving the surfactant (Poloxamer 407 or Cremophor RH 40) in deionized water and heated up to the temperature of the melted lipid phase. Afterward, the melted lipid phase was poured onto the warm aqueous phase then homogenization was started at 21,000 rpm for 10 min with homogenizer (Silent Crusher Homogenizer, Heidolph Instruments, Germany). The obtained pre-emulsion was ultrasonicated using digital ultrasonic sonicator (Model SH150-4L, MTI Corporation, Richmond, CA) for 30 min. FLZ loaded SLNs were finally obtained by allowing the hot nanoemulsions to cool to room temperature, forming lipid nanoparticles. Table 1 reports the composition of the prepared SLNs dispersion loaded with FLZ. In addition, blank solid lipid nanoparticles were prepared in a similar way without adding the drug.

**Table 1.** Composition of SLNs loaded with Fluconazole and FLZ-SLNs topical gels (% w/w).

	FLZ-SLNs dispersions(% w/w)^a^				FLZ-SLNs topical gels (% w/w)^a^				
Formulation code	Compritol 888 ATO	Precirol ATO5	Poloxamer 407	Cremophor RH40	Carpobol 934	Methyl Paraben	TEA^b^	Distilled water Up to	Gel code
SLN 1	8	–	0.5	–	–	–	–	–	–
SLN2	10	–	0.5	–	1	0.1	q.s	100	Gel 1
SLN 3	8	–	–	0.5	–	–	–	–	–
SLN 4	10	–	–	0.5	1	0.1	q.s	100	Gel2
SLN 5	–	8	0.5	–	–	–	–	–	–
SLN 6	–	10	0.5	–	1	0.1	q.s	100	Gel 3
SLN 7	–	8	–	0.5	–	–	–	–	–
SLN 8	–	10	–	0.5	1	0.1	q.s	100	Gel4

^a^ Fluconazole concentration is 1% w/w.

^b^ TEA: Triethanolamine.

### Formulation of FLZ-SLN topical gel

FLZ-SLNs dispersion was converted into gel carrier system using Carbopol (CP 934) as gelling agent. 1% w/w Carbopol 934 was dissolved in distilled water, stirred for 10 min at 1500 rpm. Subsequently, calculated amount of freshly prepared FLZ-SLNs dispersion was added and mixed for 10 min, then neutralized by drops of triethanolamine until pH 5.5. Prepared gels were further allowed to stand overnight to remove entrapped air (Kesharwani et al., 2016).

### Characterization of FLZ-SLNs

#### Determination of the entrapment efficiency percentage (EE%)

The entrapment efficiency percentage of all prepared FLZ SLNs was determined by measuring the concentration of free drug in the dispersion media. Separation of free drug from the nano lipid dispersion formulation was done by the ultracentrifugation method. Here, centrifugation of nano lipid dispersion was carried out at 14,000 rpm for 90 min using Remi cooling centrifuge (Mumbai, India). The clear supernatant from the resulting solution was diluted appropriately using phosphate buffer pH 5.5 and analyzed by ultraviolet (UV) spectrophotometer at 261 nm (Shimadzu 1800, Japan) (Agalakshmi et al., 2014). The EE% was calculated from the Equation (1) (Ahmed et al., 2014).

(1) $$\text{EE }\%\;=\;\left[\left(W_{\text{initial drug}}-\;W_{\text{free drug}}\right)/W_{\text{initial drug}}\right]\;\times \text{ 1}00$$

Where ‘*W*_initial drug_’ is the mass of initial drug used for the assay and ‘*W*_free drug_’ is the mass of free drug detected in the supernatant after centrifugation of the aqueous dispersion.

#### Particle size and zeta potential analysis

Particle size and Zeta potential was measured for selected FLZ-SLNs formulae in folded capillary cells using zetasizer (PCS, Malvern Mastersizer Hydro 2000G, UK). About 1 ml of each nanodispersions was diluted with 10 ml deionized water. Ultra sonication of the samples has been done before size determination for 5 min (Shah et al., 2012).

#### Transmission electron microscope (TEM)

The shape of the selected FLZ-SLNs dispersion was determined by the transmission electron microscope (Model JEM-1230, Jeol, Tokyo, Japan). One drop of diluted sample was placed on the surface of a carbon coated copper grid, after staining with one drop of 2% w/w aqueous solution of phosphotungestic acid. Afterward the samples were left to dry for 10 min for examination (Patel & Prajapati, 2016).

#### In vitro drug release studies and release kinetics

The *in vitro* release of FLZ from all prepared SLNs was evaluated by the dialysis bag technique (Khalil et al., 2014). FLZ-SLNs dispersion equivalent to 5 mg of FLZ were accurately weighed and transferred to cellulose dialysis membrane having a molecular weight cutoff from 12,000 to 14,000 Da. The membrane is tied with threads and immersed in 50 ml ethanol and phosphate buffer pH 5.5. The flask was kept in an incubator at 37 °C and stirred at 50 rpm, specific volume of the samples were withdrawn at regular intervals and equal volume of dissolution media was added to the release medium to replenish it. The release of FLZ from nanolipids was determined spectrophotometrically at 261 nm using prepared plain SLNs as blank. Experiments were carried out in triplicates. *In vitro* release data of FLZ from prepared SLNs were fitted to various kinetic models and were analyzed in order to explain the mechanism of drug release.

#### Differential scanning calorimetry (DSC)

The thermal characters of the prepared FLZ-SLNs were determined by DSC. In addition, DSC analyzes were performed on pure FLZ, Compritol 888 ATO and Precirol ATO5 by a Mettler Toledo DSC (Mettler-Toledo, Viroflay, France). About 1–2 mg of FLZ, Compritol 888 ATO and Precirol ATO5 have been accurately weighted in 40 μl aluminum pans. DSC scans have been recorded at a heating rate of 100 C/min and was run over the range 25–3000 °C, using an empty pan as reference (Castelli, 2005).

### Characterization of FLZ-SLNs topical gels

#### Visual appearance and pH

Visual appearance, spreadability, and clarity of prepared FLZ-SLNs gel were observed for the presence of any particular matter. The pH of each gel batch was determined using a pH meter (JENWAY 350, UK). One gram of each formulated gel was dispersed in 30 ml of distilled water, then the pH was measured which noted by bringing the electrode near the surface of the formulations and allowing it to equilibrate for 1 min (Kumar & Himmestein,^1995^; Srividya et al., 2001).

#### Estimation of drug content

One gram of selected FLZ-SLNs gel was taken into a standard volumetric flask and mixed with mixture of phosphate buffer pH 5.5: ethanol. The amount of drug per 1 g gel was determined spectrophotometrically at 261 nm after filtration through Millipore filter (0.45 μm) and the drug content was obtained from the calibration curve (Carlfors et al., 1998; Nanjawade et al., 2009).

#### Rheological studies

The viscosity of prepared FLZ-SLNs gels was studied on Brookfield viscometer (DV-II Pro Viscometer, Middleboro, MA) using CPE-42 spindle. For each sample continuous variation of the speed rate from 1 to 100 ^s−1^) then back ward from 100 to 1 s^−1^) was applied (25 ± 1.0 °C) and the resulting viscosity was measured (Wamorkar et al., 2011).

#### *In vitro* drug release studies

*In vitro* release of FLZ from different topical SLNs gel formulae was evaluated by using dialysis bag technique (Khalil et al., 2014) and performed in dissolution medium consists of phosphate buffer pH 5.5 and ethanol. FLZ SLNs gel containing equivalent to 5 mg of FLZ were accurately weighed and transferred to cellulose dialysis membrane having, molecular weight cutoff from 12,000 to 14,000 Da then the experiment completed as previous. The results were the mean of three experiments. The release profiles of FLZ were evaluated by fitting the experimental data to equations describing different kinetic orders named as zero order, first order, Higuchi, Korsemeyer–Peppas model. The accepted order of kinetics was based on high regression coefficient value.

Furthermore, the promising prepared FLZ-SLNs gel formula which based on *in vitro* release study have been chosen for further clinical evaluation on well diagnosed thirty patients of Pityriasis Versicolor in contrast to the commercial antifungal topical product Clotrimazole (Candistan 1%)^®^ Adco company.

### Clinical study of the FLZ-SLNs topical gels

#### Setting

Patient selection and recruitment were carried out in Dermatology Department, Faculty of Medicine, Tanta University Hospitals. Thirty patients with a clinical diagnosis of Pityriasis Versicolor were selected for the study.

#### Ethical approval

Written informed consent was compulsory for contribution in the study. Necessary ethical clearance was obtained from the ethical committee office code number: 89 Al-Azhar University and the study was conducted in accordance with the ethical principles that have their origin in the Declaration of Helsinki Good Clinical Practice. The staff notified the contributors with the objectives, dates, drugs, diet, possible risks, and general activities through the clinical part of the study.

#### Patients

The current study comprised thirty male patients who received a diagnosis of PV, and who had the following symptoms were eligible for inclusion: typical appearance of skin lesions and confirmed by microbiological examination. While pregnant or lactating women, those have an evidence of human immunodeficiency virus or other life threatening infection, having history of hypersensitivity to FLZ or included in any other clinical trial within the previous month will be excluded from this study. Additional exclusion criteria included any concomitant medical condition that may have presented unacceptable risks to the patient, as judged by the study investigator.

#### Study design

This is randomized prospective controlled trial with three parallel treatment arms. Eligible patients who satisfied the inclusion criteria were randomized 1:1:1 to three treatment arms according to a randomization list by closed envelope each group comprising of ten PV patients.

Group I covered the PV patients treated topically with Gel 2(1% Carpobol gel containing: SLNs of 10% Compritol 888ATO +0.5% Cremophor RH40 + 1% FLZ) twice daily for 4 weeks.

Group II comprised of PV patients treated topically with Gel 3(1% Carpobol gel containing: SLNs of 10% Precirol ATO5 + 0.5% Ploxamer 407 + 1% FLZ) twice daily for 4 weeks.

Group III (active control) consists of PV patients treated topically with commercially available product, Clotrimazole (Candistan 1%)^®^ Edco Company twice daily for 4 weeks.

In the current study, the efficiency and safety of two prepared 1% FLZ-SLNs topical gels in comparison to the commercial available topical Clotrimazole namely, Candistan^®^ cream 1% in topical treatment of PV were reported. Each patient visited the investigator four times during the trial. After the baseline visit, they were asked to report again every week for a month for follow-up to look for any relapse.

#### Assessment of efficacy and safety

Following the initial (base line) visit, the subsequent visits were scheduled on day 7, day 14, day 21, and day 30. The efficacy and safety of the prepared gels was compared with marketed Candistan^®^ 1% cream for the positive clinical response and changes from baseline for the symptoms of PV that reported every week by clinical, visual (photographic), and mycological examinations. The clinical parameters for evaluation were signs and symptoms. These parameters were assessed on a pre-determined four point scale as percentage of recovery from PV and documented by photographic pictures which show the percentage of recovery. Clinical assessment were made and recorded by three separate dermatologists.

The clinical examination findings were easily confirmed with the mycological microscopic examination of skin scales soaked in potassium hydroxide (KOH) (Thirumurthy et al., 2002; Koneman, 2006). The affected skin area was thoroughly wiped with 70% alcohol to remove the surface contaminants. After drying, the active edges of the lesions were scraped with a flame sterilized; No. 15 scalpel blade from at least two infected areas. The specimen was placed on a glass slide, one or two drops of 20% solution of KOH are added and a cover slip applied. Lactophenol cotton blue stain was added to the slide in order to examine the hyphal structure. Also, the slide briefly heated over a flame. This process allows the KOH to dissolve cellular material, leaving the hyphae and spores more easily identifiable. The slides were examined under light microscope (C x 40, 10, Olympus, Center Valley, PA). Microscopic examination demonstrated the characteristic thick-walled spherical or oval yeast forms and coarse septate mycelium, often broken up into short filaments. This combination of mycelium strands and numerous spores is commonly referred to as ‘spaghetti and meatballs’ or ‘banana and grapes’ and are characteristic of *Malassezia furfur* in PV scales (Kurade et al., 2006).

The Existing of the PV fungus, represented by the mycological microscopic examination, was categorized in three main scales as follows:

Persistence (P): still positive microscopy appearance at follow-up visits.

Persistence with improvement (PI): less density positive microscopy appearance at follow-up visits.

Eradication (E): complete microscopic disappearance of *M. furfur* in all follow up visits.

#### Statistical analysis

For clinical part: The data are expressed as the mean ± standard deviation. The statistical analysis was done using SigmaPlot^®^ 12.5 software extended with the statistical package. Two-way repeated measures analysis of variance was used to assess the significance of the difference between quantitative variables. *p* < 0.05 was considered to be statistically significant.

For mycological part: to evaluate the effect of different treatments at different weeks on the frequencies of *M. furfur* fungus degrees (P, PI, and E), pairwise comparisons were performed by Mann–Whitney U non-parametric test using IBM SPSS^®^ Statistics 20 software extended with the statistical package.

## Results and discussion

### Preparation and characterization of FLZ-SLNs

For the current study and after screening different concentration of solid lipids and surfactants by applying different methods of preparation, FLZ-SLNs were well achieved by the modified high shear homogenization and ultrasonication method as the drug showed high solubility in the molten lipids. It is a very simple and reproducible method (Wei et al., 2016).

### Entrapment efficiency (EE %)

In order to achieve high drug encapsulation efficiency, many variables were explored, including the type and concentration of both lipids and surfactants. The corresponding percentage of entrapment efficiency of FLZ-SLNs was found to be satisfactory high which is ranging from 55.48 ± 1.21 to 82.94 ± 1.24% as depicted in Table 2. This results can be attributed to the fact that FLZ is a moderate lipophilic drug (Mathy et al., 2005), and thus has affinity toward lipid matrix (Durán-Lobato et al., 2013). Moreover, this may be accredited to the structure of solid lipid used as by using highly crystalline lipids with a perfect lattice (e.g. monoacid triglycerides) lead to drug expulsion (Westesen et al., 1997). While, more complex lipids as Compritol 888 ATO and Precirol ATO5 (mixtures of mono-, di-, and triglycerides) form less perfect crystals with many defects proposing more space to incorporate the drugs (Muller et al., 2000). The results suggested that as the lipid concentration increases from 8% to 10% w/w with constant concentrations of surfactant 0.5% w/w, the drug EE increased and this results similar to finding of Thatipamula et al. (2011). This is due to an incorporation of high concentration of solid lipids leads to reduced crystallinity and increased imperfections in the crystal lattice of lipid which leaves enough space to accommodate drug molecules (Radtke et al., 2005). Also, the results showed that Compritol 888 ATO exhibited significance increase in EE than Precirol ATO5 (*p* value < .05). This may be related to the higher hydrophobicity nature of Compritol 888 ATO as it is composed of long chain fatty acids that attached to the triglycerides which lead to increase accommodation of lipophillic drugs as FLZ (Khalil et al., 2013). Moreover, it shows high drug entrapment efficiency (EE %) may be due to the existence of large amount of mono-, di-, and tri-glycerides that aids in drug solubilization (Manjunath et al., 2005).

**Table 2.** Characterizations of selected FLZ SLNs.

Formulation code	EE%^a^	Zeta potential (mv)^a^	Polydispersity index^a^	Mean Particle Size (nm)^a^
SLN1	76.72 ± 1.03	–	–	–
SLN2	82.94 ± 1.24	−21 ± 2.76	0.29 ± 0.64	307 ± 0.32
SLN3	71.27 ± 1.11	–	–	–
SLN4	80.52 ± 1.05	−33.1 ± 1.24	0.255 ± 0.47	500 ± 0.26
SLN5	71.39 ± 1.23	–	–	–
SLN6	79.03 ± 1.11	−22.9 ± 1.66	0.228 ± 0.52	292 ± 0.79
SLN7	55.48 ± 1.21	–	–	–
SLN8	77.04 ± 1.12	−25.7 ± 0.83	0.278 ± 0.79	420 ± 0.53

^a^ Values are represented as mean ± SD (*n* = 3).

Also, the results showed that there is a significance difference between the two types of surfactant (*p* value < .05) which showed that Poloxamer 407 has the higher EE for all lipids types than Cremophor RH 40 as Poloxamer 407 gave nanoparticles a smooth surface with very few or no pores to minimize the drug loss during the fabrication process (Pandita et al., 2009). In addition, the decrease in %EE between different formulations may be explained by the increased solubility of FLZ in aqueous solution when Cremophor RH 40 were used as stabilizers (Elnaggar et al., 2015).

### Particle size and polydispersity index analysis

FLZ loaded SLNs formulae that showed highest EE% subjected to further characterization of particle size, polydispersity index, and zeta potential as revealed in Table 2. The tested FLZ-SLNs formulations showed a mean particle size between 292 and 500 nm which is an optimum size for topical drug delivery (Jenning et al., 2000). FLZ-loaded SLNs, prepared with Compritol 888 ATO as the lipid matrix, resulted in larger particle size compared to SLNs prepared using Precirol ATO5, with all type of surfactants studied. This may be referred to the difference in the viscosity and chain length of the utilized lipids (Ahlin et al., 1998). Furthermore, this may be related to the melting point of the lipid, as Compritol 888 ATO has a higher melting point (m.p. 69.0–74.0 °C) than Precirol ATO5 (m.p. 50.0–60.0 °C), which lead to delay lipid crystallization from the hot homogenized circumstance causing growth in the particle size (Ekambaram & Sathali, 2011). These results were in agreement with studies on cyclosporine-loaded SLNs achieved by Gokce et al. (2008). Moreover, Khalil et al. (2014) concluded that Compritol 888 ATO and Precirol ATO5 resulted in the largest and smallest particle sizes, respectively, regarding the investigation of the preparation of meloxicam-loaded lipid nanoparticle-based hydrogels for topical application.

FLZ-SLNs dispersion exhibited lower particle size when prepared using Poloxamer 407 as stabilizer than the other surfactant regardless the lipids used. This result may be due to Poloxamer 407 have higher molecular weight and higher HLB value when compared to other used surfactant Cremophor RH40 (Jawahar et al., 2009). The polydispersity index (PDI) is a marker of particle size distribution. The PDI values were lower than 0.3 for all the formulations regardless of the surfactant, lipid type. This indicates narrow size distribution which reflects the suitability of the method of preparation (Bahari & Hamishehkar, 2016).

### Zeta potential analysis

Zeta potential is an important surface characterization technique which helps in determining the potential stability and surface charge of nanoparticulate system. Usually large negative or positive zeta potential value required for SLNs dispersion stability as electrostatic repulsion between particles with same charges avoids aggregation of particles (Manjunath et al., 2005). Zeta potential data for tested FLZ-SLNs dispersions are shown in Table 2. Despite the slight differences in zeta values -21 mV to −33 mV, all FLZ-SLNs formulae are negatively charged which assures a good physical stability of the formulation by electrostatic repulsion, avoiding the occurrence of particle aggregation or coalescence. These results were in agreement with studies on lopinavir solid lipid nanoparticles obtained by Negi et al. (2013).

Zeta potential was slightly more negative in the SLNs prepared with Cremophor RH40 compared with the SLNs prepared with Poloxamer 407, this may be due to the chemical nature of Poloxamer 407 that act as a steric stabilizer and decreases the zeta potential due to shift in the electric shear plane of particles (Kamboj et al., 2010).

### Electron microscope examination

TEM photographs (data not shown) were taken to corroborate SLNs size and assess SLNs morphology. This revealed that all prepared formulae were found to be spherical in shape with a smooth surface and colloidal sizes with no aggregation appeared as black dots and uniformly dispersed in the media. This indicates that the employed method of preparation ‘hot homogenization method’ was successfully achieve SLNs systems with uniformly distributed particles and with size range of nanometers and these results are in agreement with findings of Ramasamy et al. (2012).

### *In vitro* drug release and kinetics study

**Figure 1.**
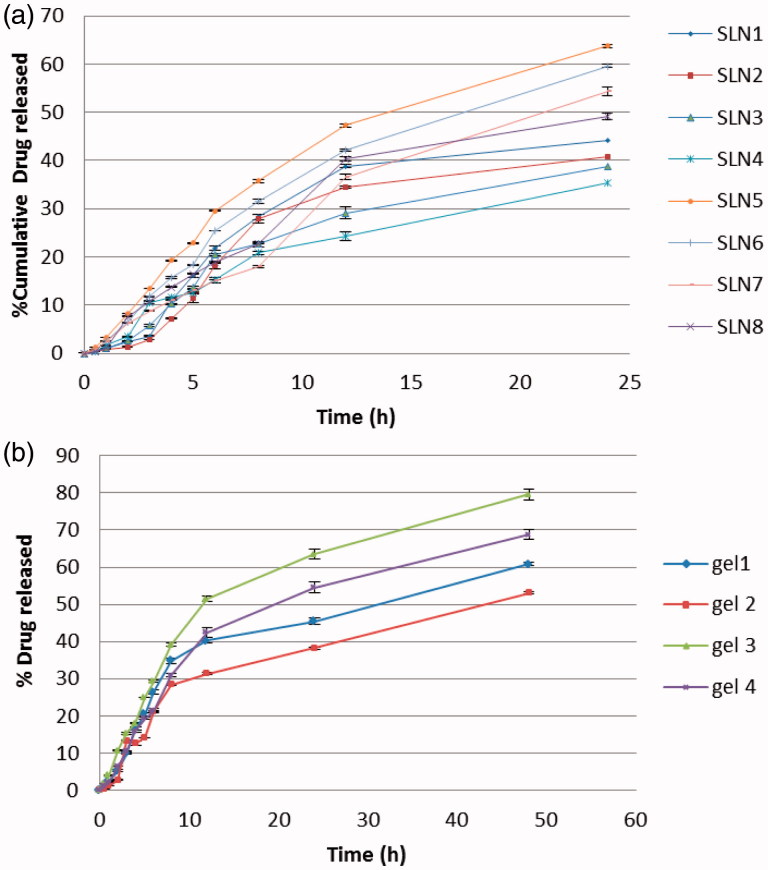
*In vitro* release profiles of FLZ from (a) prepared SLNs and (b) relevant SLNs gels.

*In vitro* drug release from the SLNs of FLZ was performed in PBS (pH 5.5 at 37_ _°C) using dialysis bag technique over 24 h and the cumulative release percentages are illustrated in Figure 1(a). Data of the release profiles evidences that developed SLNs were capable to release FLZ in controlled mode as the cumulative release percentage of FLZ over 24 h ranged from 32% to 63%. The slow release of the FLZ from most prepared SLNs suggests homogeneous entrapment of the drug throughout the systems, Paliwal et al. (2009) explained similar concept and stated that controlled drug release can be obtained when the drug is evenly dispersed in the lipid matrix. Remarkably, the type of surfactant showed a great impact on the FLZ release pattern from prepared SLNs. Poloxamer 407 showed a significant increase (*p* value < .05) in drug release rate from SLNs when compared with Cremophor RH40. This could be due to the higher HLB value of Poloxamer 407 (18–23) than the other surfactants used as stabilizers Cremophor RH 40 (14–16) (Ekambaram & Sathali, 2011). In addition, the Poloxamer 407 has surface activity so it reduces the interfacial tension between the SLNs and dissolution medium and decreases the aggregation of drug particles and enhances the dissolution rate of drug (El-Badry et al., 2013). Regarding the type of lipid, Compritol 888 ATO produced SLNs with larger particle size; higher drug entrapment and significantly (*p* value < .05) more prolong FLZ release than Precirol ATO5. This finding was in consistent with that observed by Abdelbary & Fahmy (2009) when they used Compritol 888 ATO and Imwitor 900K as lipid component in studying the effect of different concentrations of lipids on the drug release pattern. This may be related to the triglycerides with lipophilic long chain fatty acids which prolong the release of lipophilic drug (Hackett et al., 2013).

Moreover, the difference in lipid melting points may lead to difference in the release profiles of drugs as indicated by Paolicelli et al. (2009). As Precirol ATO5 has lower melting point than Compritol 888 ATO, so that at the release experiments temperature it shows higher mobility consequently greater cumulative drug release. On the other hand, the results indicate the impact of lipid concentration on FLZ-SLNs release pattern regardless the lipid type. As by increasing the concentration of lipid from 8% to 10% w/w resulted in corresponding decrease in the drug release. This can be attributed to the higher lipid content encapsulating the drug, thus reducing drug partition in the outer phase and consequently its release in the receiver media. This result was in agreement with findings of Khalil et al. (2013). In another words, as the lipid concentration in the SLN preparation was increased, the thickness of the lipid coating increased thereby increasing the length of diffusion resulting in decrease in the drug release as shown in Figure 1(a) (Pandita et al., 2009).

Furthermore, *in vitro* release profiles for the selected prepared SLNs were applied on various kinetic models (zero order, first order, and Higuchi equations). In order to figure out the mechanism of drug release, the rate constant as well as the highest correlation and the best fitted line was obtained. The release pattern of the drug from almost all SLNs formulations were best fitted into Higuchi equation that describes the diffusion of drug from homogenous and granular matrix systems (Vivek et al., 2007) with some fitting to zero order equation. This explains why the drug diffuses at a slower rate as the distance for diffusion increases, which is referred to as Higuchi’s kinetics, i.e. matrix diffusion controlled mechanism. Considering these observations it was concluded that all the batches of SLNs had the potential for sustained drug delivery. This finding is in a good agreement with the previous studies (Tiyaboonchai et al., 2007; Vivek et al., 2007).

### Differential scanning calorimetry (DSC) of FLZ SLNs

DSC is frequently used in the pharmaceutical field as a thermal analysis technique, to provide information about drug-excipient incompatibility in the formulation (Naidu et al., 2004). DSC curve of pure FLZ powder, Figure 3 showed a sharp endothermic peak at 137.05 °C corresponding to its melting point, indicating its characteristic crystalline nature. Bulk Compritol 888 ATO showed sharp melting peak at (72.24 °C) confirming the presence of the stable β/form, while Precirol ATO5 at (56.41 °C) which indicated the absence of traces of impurities as shown clearly in Figure 3. It is worthy to say that DSC curves revealed that all FLZ-SLNs formulations possess melting point over 40 °C which indicates the solid state at room temperature (Nayak et al., 2010). The thermograms of all examined SLNs formulae showed the absence of characteristic melting peak of FLZ around (137.05 °C) as (data not shown) concerning SLN2 (FLZ-Compritol 888 SLNs) and SLN7 (FLZ-Precirol ATO5 SLNs) thus suggesting that no free drug crystals were reported in the systems. Moreover, this may indicates the transformation of FLZ from crystalline to the amorphous form upon loading into SLNs which may be attributed to complete solubilization of the drug in the lipid matrix or molecularly dispersion of the drug in the lipid matrix (Ramasamy et al., 2012). These findings confirm the slow release profile that we achieved in this study as the lack of crystallinity suggests better drug dispersion and increased drug–matrix interactions leading to the slow release kinetics of drug from the matrix (Mahato, 2007).

Furthermore, a small increase on the onset and on the melting temperature of the lipid when it is mixed with drug was observed. These phenomena were previously described by Müller et al. (2008). It is worthy to note that the melting enthalpy values of all SLNs were lower than those of the initial lipid (data are not presented), indicating an increased number of lattice defects in the developed FLZ-SLNs resulting in formation of amorphous regions in which the drug is located. These results were in agreement with findings of Prasad et al. (2010) and Mehnert & Meader (2012). This may be explained as less energy is required for melting less ordered crystals or amorphous solids than crystalline substances which need to overcome lattice forces as (Pople & Singh, 2006).

### Preparation and characterization of FLZ SLNs topical gel

According to the best result of EE% only four formulae of FLZ-loaded SLNs incorporated into gel formulation using 1% Carbapol 934 as gelling agent and followed by several characterizations as:

### Visual appearance, pH, and drug content

All the prepared gels of selected FLZ-SLNs formulae were spreadable on the skin surface, white, smooth, homogenous with semisolid consistency, and show no synerisis. The pH of all prepared FLZ-SLNs gels was found to be 5.5–6.7 with drug content in range 8–9 mg/1 g of gel. These properties give the prepared gels the suitability for topical administration (Kaur & Guleri, 2013).

### Rheological study

The rheological properties of lipid nanoparticles affect their potential for dermal application in a fundamental way (Lippacher et al., 2002; Muller et al., 2002). Therefore, the rheological behavior of gels loaded with FLZ-SLNs for topical drug delivery was evaluated. The rheograms of all FLZ-SLNs gels (data are not shown) revealed non-Newtonian flow behavior with no constant viscosity (Barnes, 1997; Illinga and Unruh, 2004; Liu et al., 2008). The flow behavior of FLZ-SLNs gels was also characterized by shear-thinning properties with variable thixotropy as by increasing the shear rate the viscosity of gel consequently decreased (Khalil et al., 2014). The combined shear thinning behavior and thixotropy are desirable characteristics for topical formulations, as they facilitate processing during manufacture and the flow from the container, and improve spreading on the skin. In addition, the applied film can gain viscosity instantaneously and thus resist running (Ammar et al., 2011; Sheth, 2007).

### *In vitro* drug release studies

The release of FLZ from different lipid nanoparticles gels was performed using the dialysis bag membrane (Pandurangan et al., 2016) and the results are presented in Figure 1(b). The results revealed that lipid nanoparticles gels were able to release FLZ in controlled manner where the percentage of drug released ranged from 53% to 83% after 48 h. Comparing the drug release profile of nanoparticle dispersions and their corresponding gels, the release of FLZ from the investigated gel formulations at the end of 48 h was slower than that of their nanoparticles dispersions. Incorporation of nanoparticulate dispersion into gels further decreased the drug release. This result was probably due to the release-retarding effect of the polymeric matrix of gelling agents (Pople and Singh, 2006). As shown in Figure 1(b), results showed that the highest drug release was related to gel 3 and gel 4 which composed of Precirol ATO5 as lipid content followed by Compritol 888 this is due to the highest mono glyceride content and the lowest melting point of Precirol ATO5 lipid which may lead to a greater mobility at the temperature used in the release experiment. Also, the results showed that Poloxamer 407 surfactant showed the highest release than Cremophor RH40. In addition, all investigated gels demonstrated prolonged release characteristics following Higuchi kinetics. These results suggested that the primary release mechanism of FLZ from lipid matrix and gel is diffusion (Muller et al., 2000; Pople & Singh, 2006).

### Clinical study

The clinical study was performed to evaluate two formulae of prepared FLZ-SLNs topical gels which have the lowest (50%) and highest % (80%) drug release from gel matrix naming gel 2 and gel 3, respectively. In addition to compare the result with market product Candistan^®^ cream. Thirty patients were successfully enrolled for 4 weeks in this study to evaluate the treatment of PV. Diagnosis was done clinically based on the typical appearance of skin lesions and confirmed by microbiological examination. Each 10 patients enrolled randomly in group and were treated with prepared gel 2 or prepared gel 3 or market product Candistan^®^ cream for group I, II, and III, respectively. The treatments were applied twice daily for 4 weeks. The volunteers in each group were followed up by clinical and mycological examination every week during the therapy. Furthermore, baseline photographs were taken to the affected areas of every patient to document the pretreatment skin lesion and after week two and four of starting therapy to evaluate the clinical efficacy and safety of different treatment regimen.

**Figure 2.**
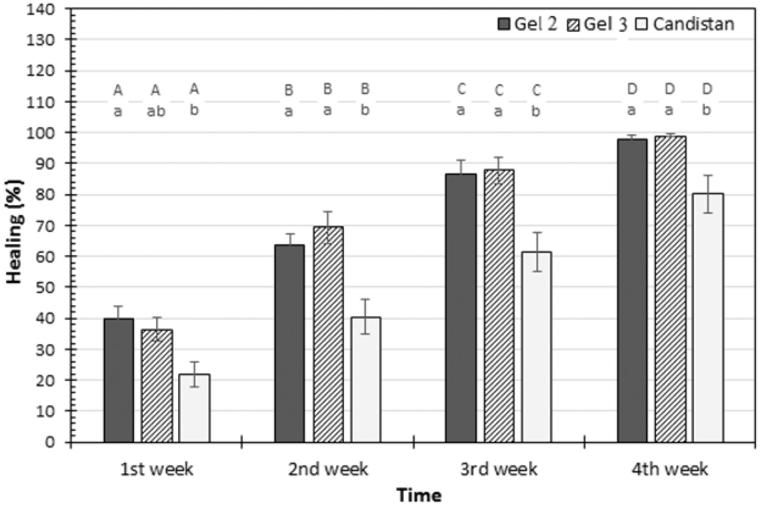
Mean of healing percentage measured in patients at first, second, third, and fourth week of different treatments application. Capital letters represent the significance of same treatment at different weeks. Small letters represent the significance between different treatments within the same week. Error bars represent standard error (SE).

The volunteers showed good acceptance of the formulations and reported no adverse reactions while using them or afterwards. From Figure 2, statistical analysis for clinical examination showed that there was a highly significant difference (*p* < .05) in percentage of cure from PV among all groups treated with gel 2, gel 3, and market product Candistan^®^ before and after treatment. The results revealed that the percentage of healing from fungal infection along the four weeks for gel 2 ranged from 40% to 98%, for gel 3 ranged from 36% to 99% these results in agreement with Karakaş et al. (2005) who found that 75% patients showed a complete cure from PV after 4 weeks treatment with oral FLZ. While the percentage of relief from fungal infection after four weeks application of Candistan^®^ cream was ranged from 22% to 80%. From these percentages we can conclude that there are significance difference between gel 2, gel 3 and commercial Candistan^®^. Furthermore, by comparing the results of healing rate between group I and group II, the results showed that there is no significance difference (*p* > .05) between group I (treated with gel 2) and group II (treated with gel 3).

**Figure 3.**
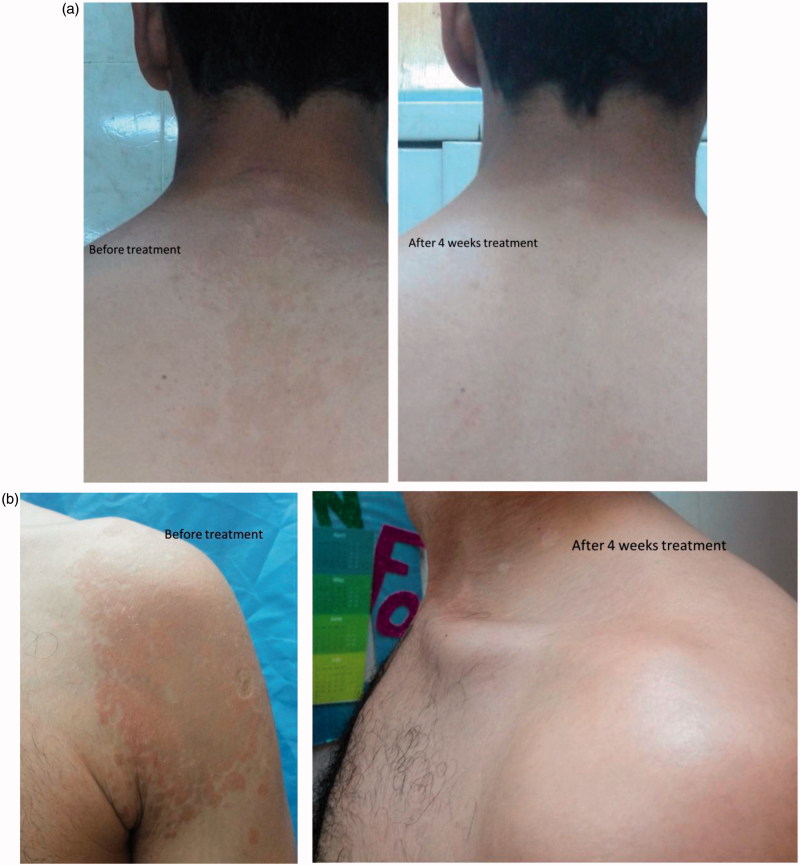
Photographic pictures before and after treatment with (a) gel 2 and (b) gel 3.

Moreover, the clinical improvement established upon taking photographic pictures for patients infected with PV before and after treatment with gel 2 and gel 3. The results showed that there are great improvements of treatment from fungal infection during 2, 3, and 4 weeks of treatment (Figure 3).

Regarding the mycological culture, existing of the *M. furfur* fungus represented by the mycological examination was characterized in three main scales as follows:

Persistence (P): still positive microscopy appearance at follow up visits,

Persistence with improvement (PI): less density positive microscopy appearance at follow up visits,

Eradication (E): complete microscopic disappearance of *M. furfur* in all follow up visits.

**Figure 4.**
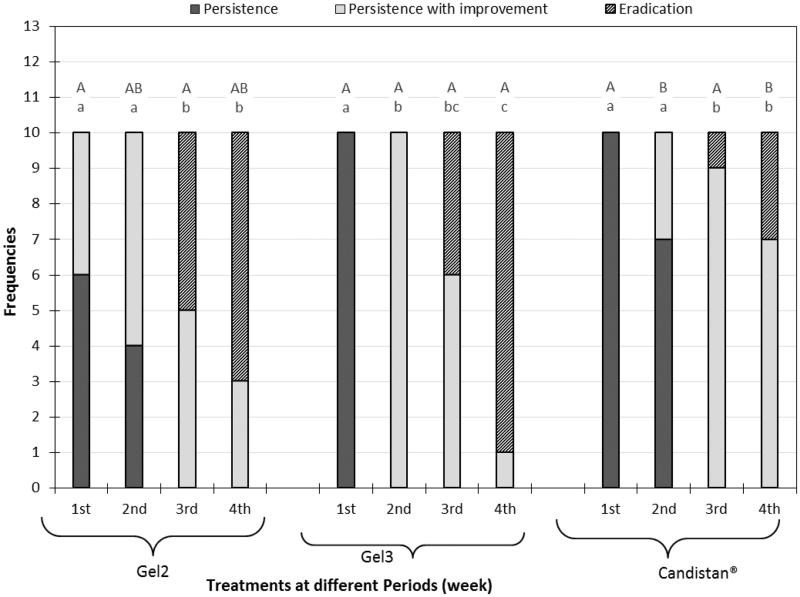
Mycological results representation of gel 2, gel 3, and Candistan^®^ cream with pairwise comparison at each weak; capital letters represent the significance of same treatment at different weeks and small letters represent the significance between different treatments within the same week.

It is obvious from Figure 4 that the percentage of cured cases from the start to the end of the study was significantly larger (*p* < .05) in group I and II than in group III. It is worthy to say that group II show greater effect in percentage of eradication from *M. furfur* fungus than group I but this difference was non-significance (*p* > .05). This may be attributed to the higher drug release from SLNs as confirmed by the *in vitro* release findings in the previous part of this study. In group I, at the start of the study all the patient were infected with PV, at the end of experiment 70% of patient showed complete eradication and 30% showed persistence with improvement from fungal infection. While in group II, at the start of the study all the patient were infected with PV, at the end of experiment 90% of patient showed complete eradication and 10% showed persistence with improvement from fungal infection.

However, in case of group III, at the start of the study all the patient were infected, at the end of experiment only 30% of patient showed complete eradication and 70% showed persistence with improvement from fungal infection.

To the best of our knowledge, there is no clinical study regarding efficacy of topical FLZ for the treatment of PV. However, it was proven in many studies that FLZ is highly effective in treating PV when taken orally (Farschian et al., 2002; Karakas et al., 2005; Yazdanpanah et al., 2007). Although the application of oral antifungals can shorten the duration of treatment of PV (Gupta et al., 2002), they are considered second line treatment as they can be associated with serious adverse events as hepatotoxicity associated with oral ketoconazole (Gupta et al., 2015; Yan et al., 2015).

In our study, topical FLZ seems to be faster and more effective than topical Candistane^®^ in the cases of PV. Most of the cases that were treated by topical FLZ-SLNs gels seemed to be satisfied with the quick cure related (after 1 week application) to commercial topical antifungal therapy with average 40.0 cure rate in gel 2 formula and 36.5 cure rate in gel 3 formula while that of Candistan^®^ was 22.0. In addition, there was no irritation or significant side effects with the topical FLZ formulations.

Shahid et al. (2000) study stated that the cure rate (clinical and mycological cure) of PV ranged between 78 and 98% on the fourth week follow up when 300 mg of FLZ was given orally once weekly for 2 weeks . Our treatment results were close to these findings, as we achieved clinical cure ranged between 98 and 99% and mycological cure ranged between 70 and 90% on the fourth week of treatment, although our treatment was applied topically. In addition, the compliance of the patients was good, and no side effects were observed. These results may suggest that topical FLZ in the form of SLN-loaded gel can provide immediate reductions in infectivity, and free of systemic adverse effects. The efficiency of FLZ is believed to be related to the fact that it is delivered to the stratum corneum in high concentrations which is important factor in the treatment of PV (Haneke, 1990). However, these results in contravene with Banerjee et al. (2012) who found that 0.5% FLZ gel is comparable to 1%clotrimazole cream when used topically in tinea corporis. This is may be attributed to that PV is caused by more superficial infection usually at stratum corneum caused by Malassezia while other types of tenia are deeper infections which involve the dermis. In addition, enhanced drug penetration might be related to the nanometer-sized SLNs because the highly specific surface area facilitates contact of the encapsulated drug with the stratum corneum and may favor accumulation for several hours, which allows for sustained drug release (Jenning et al., 2000).

The majority of studies to date suggest that nanoparticles only permeate the superficial layers of the skin in vivo and remain in the stratum corneum (Watkinson et al., 2013). Therefore, an enhanced bioavailability of the encapsulated material to the skin is achieved. SLN enhance the penetration and transport active substances particularly lipophilic agents and thus intensify the concentration of these agents in the skin (Muller et al., 2002).

This is may be also attributed to that the nanoparticles show a high affinity for cellular membrane mainly due to electrostatic interactions (Bernfild et al., 1999). Furthermore, SLN may also offer improved drug penetrating through the stratum corneum due to the better occlusion of the nanoparticles compared to traditional creams and gels (Wissing & Müller, 2002).

Nanostructured lipid carriers NLC also offer possibilities in the treatment of significant diseases as psoriasis which was confirmed by acitretin loaded NLC in a clinical study (Agrawal et al., 2010). Several studies that investigate the efficacy of antifungal agents for treatment of human mycoses when incorporated with SLN concluded that when the drugs were incorporated into an SLN there was an increased rate and level of skin penetration, higher efficacy, and less local side effects (Jessica et al., 2013).

Sustained release of FLZ and higher skin penetration were possible explanations for the enhanced therapeutic efficacy and increased tolerability of FLZ-SLNs gel in comparison to conventional marketed Candistan^®^ cream for topical treatment of PV.

## Conclusion

PV is one of the most common cutaneous dermatologic conditions worldwide. As Malassezia species are endogenous to the skin flora, this condition is particularly difficult to eradicate. In the meantime, there are a number of topical and oral antifungal treatments that are effective in improving clinical symptoms and inducing mycological cure. However, topical therapy is the first line of treatment for PV as they are fast-acting and well tolerated. Based on the accumulated evidence, FLZ as antifungal agent for treating PV is only available as oral therapy. Therefore, in this attempt, we successfully developed topical gel of FLZ loaded in SLNs prepared by hot homogenization technique followed by carbopol gelation. Developed FLZ-SLNs were characterized and assessed for particle size, zeta potential, entrapment efficiency, *in vitro* drug release pattern, TEM, and DSC. In addition the optimized FLZ-SLN gels were subjected to randomized, controlled clinical trial to evaluate the efficacy of topically applied FLZ in the treatment of PV. Clinical investigations have demonstrated the superior clinical efficacy of prepared topical FLZ-SLNs gels (Gel 2 and Gel 3) in contrast to marketed product Candistan^®^ in treating PV. The developed FLZ-SLNs gel exhibited improved skin penetration due to enhanced contact between FLZ and skin resulting from the large particle surface area and film formation.

`Relapse is a widespread concern and a likely possibility. Consequently, future work on the prepared FLZ-SLN topical gel would be promising with respect to evaluation of effectiveness as prophylactic treatment as well as detailed regimen (dose and duration) study which will be the forefront of our laboratory in order to be transformed into potential marketed product.
