# Correction to: El-Housiny et al., Fluconazole-loaded solid lipid nanoparticles topical gel for treatment of pityriasis versicolor: formulation and clinical study

**DOI:** 10.1080/10717544.2017.1419584

**Published:** 2017-12-20

El-Housiny S, Eldeen MAS, El-Attar YA, Hoda A, Salem HA, Attia D, Bendas ER and El-Nabarawi MA. (2017). Fluconazole-loaded solid lipid nanoparticles topical gel for treatment of pityriasis versicolor: formulation and clinical study. *Drug Delivery*, Vol. 25 No. 1, pp. 78–90. https://doi.org/10.1080/10717544.2017.1413444.

When the above article was first published online, there were errors in the affiliation of the author Bendas ER and in the captions of Tables 1 and 2.

The corrected captions are:

**Table 1:** Composition of SLNs loaded with Fluconazole and FLZ-SLNs topical gels (% w/w).

**Table 2:** Characterizations of selected FLZ SLNs.

And the corrected affiliation for the author Bendas ER is:

Department of Pharmaceutics and Industrial Pharmacy, Faculty of Pharmaceutical Sciences and Pharmaceutical Industries, Future University in Egypt, Cairo, Egypt

These have now been corrected.

